# Mapping the little brain at the heart by an interdisciplinary systems biology team

**DOI:** 10.1016/j.isci.2021.102433

**Published:** 2021-05-05

**Authors:** Rajanikanth Vadigepalli, James S. Schwaber

**Affiliations:** 1 Daniel Baugh Institute for Functional Genomics/Computational Biology, Department of Pathology, Anatomy, and Cell Biology, Thomas Jefferson University, Philadelphia, PA, USA

**Figure:**
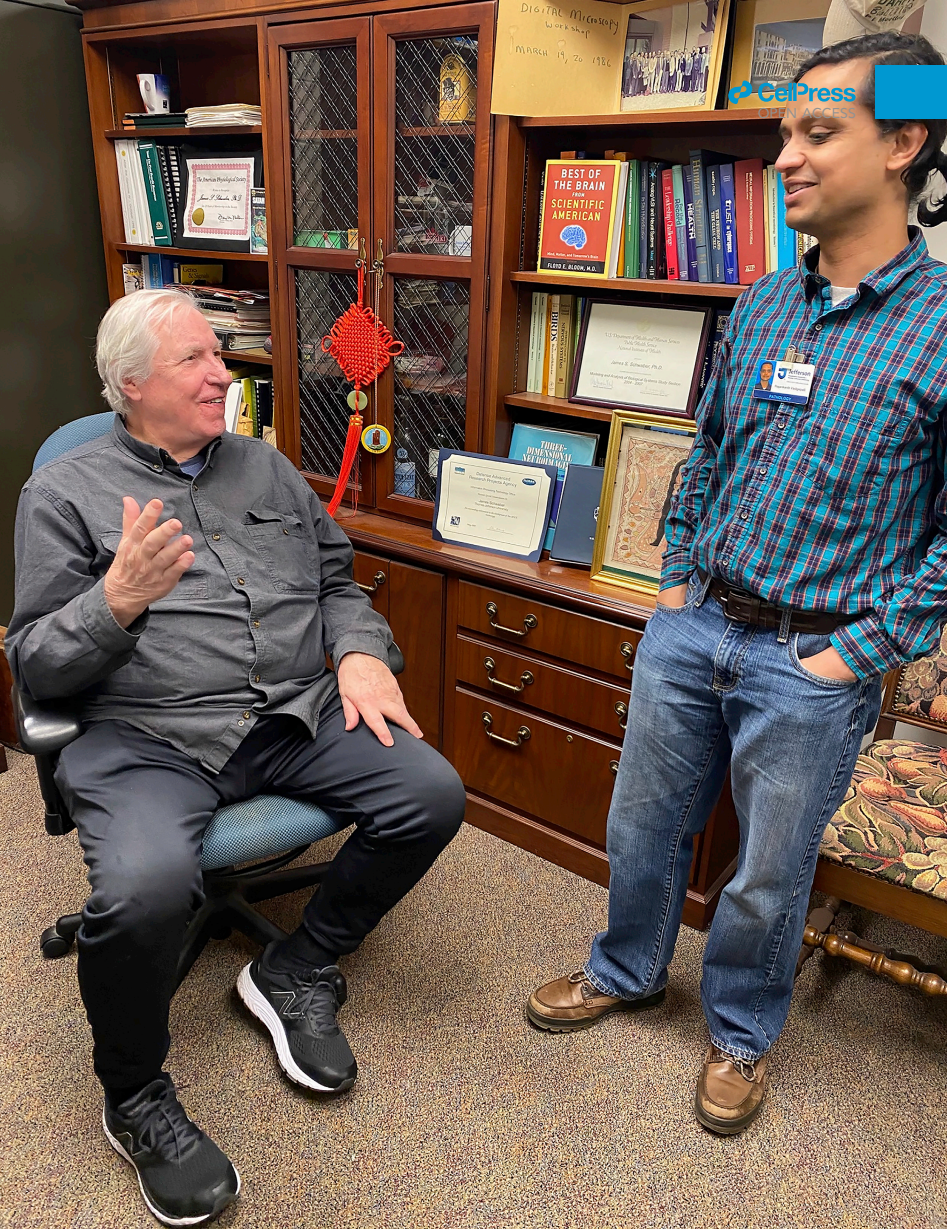
James S. Schwaber (left) and Rajanikanth Vadigepalli (right) have been at the core of an extensive project centered at Thomas Jefferson University that has brought together teams from many disciplinary backgrounds across academia and industry set on mapping the ICN system.

Heart disease is the leading cause of death worldwide. It is known that the neurons located at the heart (sometimes called “the little brain at the heart”) are a crucial player in maintaining heart health. The latest research efforts have been aimed at uncovering how one can understand the finer details of the cardiac nervous system and improve heart health in disease. These efforts have been set back by the lack of a comprehensive anatomical, molecular, and physiological understanding of the heart's nervous system. One of the initial goals of the NIH Common Fund Program Stimulating Peripheral Activity to Relieve Conditions (SPARC) was to develop such a foundational resource upon which new research studies could be initiated for developing maps and tools to identify and influence therapeutic targets.

The collaboration that produced our comprehensive mapping of the intrinsic cardiac nervous (ICN) system and model has a long-standing core and an ad hoc network that came together to result in the work published in *iScience***,** “A Comprehensive Integrated Anatomical and Molecular Atlas of Rat Intrinsic Cardiac Nervous System” (https://www.cell.com/iscience/fulltext/S2589-0042(20)30325-4).

This collaboration's enduring core is a partnership of James Schwaber, a neuroscientist, and Rajanikanth Vadigepalli, a chemical and process control systems engineer. The two of us have pursued a complementary collaboration on the relationship between peripheral organs' activity and the visceral-emotional neuroaxis for decades. The present work also required Zixi (Jack) Cheng, a former collaborator with experience mapping the heart's innervations. Also, two industrial collaborators brought unique technical expertise and resources: Susan Tappan, Maci Heal and MBF Bioscience for the 3-dimensional mapping software & graphics, and Navid Farahani and his team at 3Scan (now Strateos) for section automation & high-spatial resolution imaging for the whole heart. At Jefferson, we recruited several trainees (Jonathan Gorky, an MD-PhD student; Alison Moss, a biochemistry graduate student) and technicians (Shaina Robbins, a biotechnology graduate; Sirisha Achanta, a biomedical sciences graduate) across disciplines whose dedicated diligence was necessary to troubleshoot and advance the project.

## Proximity

### What was the motivation to launch your interdisciplinary project?

The present interdisciplinary project and publication roots go back to the early 1980s when J.S.S was drawn out of academia and into the DuPont company to pursue opportunities for interdisciplinary collaboration with molecular biologists and engineers. In the fullness of time, this led to a funded National Science Foundation grant with one of the engineering postdocs, Francis J. Doyle III (now Dean of Harvard Engineering and a member of the National Academy of Engineers), as he was just starting his first faculty appointment. Together, they recruited to their project a postdoc, Z.C. (now on the faculty at the University of Central Florida) and a graduate student, R.V. (now on the faculty at Thomas Jefferson University), who were imperative to the research effort as reflected in the present paper. While their experimental and modeling study of the ICN system suggested novel circuitry and potential nonlinear dynamics, the true testing of those insights awaited new technological advances that enabled high-resolution anatomical and molecular assays.

Four years ago, the same core group was drawn together again and was excited to find the NIH Common Fund Program SPARC. The SPARC program's goals aligned so strongly with our own long-standing interests and collaborative efforts and promised to fill a massive hole in existing funding and research activity relating to the vagus nerve. Given that the vagus nerve activity regulating the heart is crucial to heart health, this systems-level multiorgan focus has been a long-standing deficit in NIH funding priorities.

### What are the key factors that stimulate interdisciplinary research in the context of your research?

*Our systems biology approach is increasingly enabled by rapid advances in genomic scale acquisition of broad, quantitative data sets.*

The key factor is a “systems biology” point of view that can connect the relevant technical and wet lab disciplines to provide an access point for each field. By systems biology, we mean to study the functions and diseases not just from a granular level of mechanism and fixed-point causes but rather from interactions of many players in multidimensional networks across scales. Our systems biology approach is increasingly enabled by rapid advances in the genomic-scale acquisition of broad, quantitative data sets.

## Language

### What were the challenges in communication between disciplines, and how did you solve them?

We began the project before COVID-19 through in-person discussion and established common ground by focusing on the intersection of interests of people across disciplines to achieve shared goals. For example, we visited 3Scan/Strateous in San Francisco to observe the technology and its limits. We visited MBF in Vermont to outline software goals. Then S.T. from MBF provided an all-day hands-on tutorial on the MBF software and its applications. Together with SPARC MAP-CORE collaborators, we worked out a consensus of ontological terms to map the large-scale data and communicate the processed and annotated information, which is challenging.

Having said all that, we found that this collaboration was quite hard. The complexity and cutting-edge technological aspects of each team's work required strong communication to keep us aligned and coordinated. We struggled with what we came to call “slippage” where there was a tendency over time to lose alignment of objectives and activities key for overall project deliverables. Online project management tools were some help, particularly “Scrum”, but frequent conversations were crucial for realignment. During the project, bringing together so many moving parts with different outlooks was quite challenging while structuring the manuscript and developing ways to present ideas in figures. The key that got us through was to keep realigning with the overall vision of what we hoped to contribute to the field.

### What are the main challenges you [faced so far/project for the future]?

Access to cutting-edge technology drives industrial and academic interaction. It may be the case that when a valuable new technology emerges, the opportunity to develop it lies in a commercial startup, not within the resources of a typical lab. As it turned out, the opportunity had a short time window during which the industrial partner had the bandwidth to engage and contribute to the large-scale effort as their priorities can shift dynamically in response to market opportunities. To meet this goal, we prioritized our needs from different teams in image acquisition, processing, annotation, cross-checking, and mapping to molecular data that all had to be pipelined, keeping this bandwidth constraint of the industrial partners front and center to drive weekly priorities. Working with collaborators has also led to the challenge of having to resolve understanding scientific advances in the framework of deadlines and project objectives.

## Research methods

### Describe your approach to develop or adjust the methodology for the needs of interdisciplinary research

The genome project unleashed an abundance of joint engineering-biology ventures that allows high-throughput data acquisition, better computational data management, and systems biology for fundamental and clinical research. There is a choice researchers can make in pursuing standalone projects with a focus on a specific mechanism or instead take a “bird's eye” overview of the field. Both approaches are valuable, but our research falls more into the latter. One way to describe our research is trying to “predict and control” these mechanisms from the desired endpoint, unlike the traditional route to “understand, and describe THE mechanism.”

In doing so, we face two decisions: (1) decide on the most impactful “target of opportunity” and (2) since currently, we have rapidly emerging technology at our fingertips, we need careful consideration to apply the optimized technology for our problem and biological question. An example of our misjudging the second decision is that we thought that, because we had microfluidic high-throughput qPCR working for single cells acquired by laser capture microdissection, we could extend this approach to RNA-seq. This would permit both precise anatomical mapping of each cell with greatly expanded transcriptomic data. In practice, this proved a steep climb, and it required massive time and effort to acquire the “sweet-spot” compromise reflected in the final manuscript.

### How do you prepare your students/researchers for interdisciplinary research methods?

We cross-train students and give them projects that involve them in every stage of the work. For example, engineers who last took biology in 10th grade take the first-year graduate courses and some medical courses required for the biology grad students (and typically rise to the top of the class). The biology grad students take bioinformatics, statistics, and data visualization courses to gain proficiency with high-throughput data management and analysis, including learning how to write code in the R-statistical package, Python, and MATLAB. We find engineers should learn the wet-bench procedures to be able to know the limits in the experiments they design. The biologists learn to analyze their data and provide analytical results to describe its significance. Overall, having ownership and independence with the entire process forces a deeper understanding of the discovery process and permits feedback from one part of the process to readily influence the others.

## Governance

### What is your strategy to secure and maintain funding of interdisciplinary projects?

Our approach is to be opportunistic, particularly to DARPA and NIH FOA that explicitly aim to fill an interdisciplinary gap. Although some funding architectures may look more for traditional field-specific projects to fund, we still find that we can search for the right fit for interdisciplinary projects. As the NIH has invited interdisciplinary programs, there has evolved, in parallel, guidelines and protocols for multi-PI management, including conflict resolution, that form part of the review/critique of an application. So, there is a directional orientation to promote collaborative studies. That said, our experience has been that it remains extremely difficult to secure and sustain funding outside of NIH Common Fund initiatives for large-scale interdisciplinary projects that integrate efforts from industry and academia. Going forward, we believe that our success will depend on applying to those unique funding opportunities that explicitly seek interdisciplinary work.

### How do you plan the development of your (or your student's/postdoc/ECR) interdisciplinary career in the long term?

*Interdisciplinary science remains rare and finding “homes” that will nurture the students and support their approaches in team science has to be approached with care.*

We are finding our way on this; interdisciplinary science remains rare, and finding “homes” that will nurture these students and provide an encouraging support system is essential. In many ways, industry, for example, pharma and biotech, startups offer an excellent and much appreciated career path for cross-trained scientists. Currently in academia, it seems the importance of these views is increasing, but there remain several structural roadblocks in nurturing young scientists along these interdisciplinary paths and collaborations, instead of focusing solely on independent contributions. Overall, incorporating collaboration early in a scientist's career is beneficial over having them start their careers isolated in their search for independence. Growing independence while engaging in collaborations does not have to be at odds with each other.

## Publication

### How do you decide on the publication strategy for your interdisciplinary studies?

Our broad experience in publishing over the past several years has helped us to sensitize the critical impact of a journal's mission and the editorial intent to promote interdisciplinary work in finding a suitable match. *iScience* seems to us as a visionary of how we would hope science evolves, changing to team approaches and multidisciplinary efforts.

In addition, the NIH SPARC Program Officials take a very involved, “hands-on” approach to managing SPARC projects. Our impression is that they prioritize getting results in the press as rapidly as possible (e.g. prioritized over impact factor, as the review process for the latter can be extremely long and uncertain), including making results freely available immediately on the SPARC Data Portal. The online *iScience* requirements and rapid review process fit these goals.

## Final thoughts

### What tips would you give to anyone considering following an interdisciplinary career?

*Do not underestimate the challenges and frequent need to help others recalibrate how meaningful scientific progress is made in teams.*

Do not underestimate the challenges and frequent need to help others recalibrate how meaningful scientific progress is made in teams. Be prepared to take some slings and arrows from the “established” norms of academic processes. The normal evaluations for promotion and tenure may radically undervalue a faculty member of a research team, for example. But the trade-off is that you are not boxed into the confines of a long-established problem and approach. Rather you are wildly empowered to reconceive large issues and opportunities and have the technical and intellectual capability to take advantage of the revolution sweeping over biomedical science.

